# Soil Microbial Community Characteristics and Influencing Factors in Alpine Marsh Wetlands with Different Degradation Levels in Qilian Mountain National Park, Qinghai, China

**DOI:** 10.3390/biology14060598

**Published:** 2025-05-24

**Authors:** Jintao Zhang, Xufeng Mao, Hongyan Yu, Xin Jin, Lele Zhang, Kai Du, Yanxiang Jin, Yongxiao Yang, Xianying Wang

**Affiliations:** 1 Key Laboratory of Physical Geography and Ecological Conservation of the Qinghai-Tibet Plateau, Key Laboratory of Physical Geography and Environmental Processes of Qinghai Province, Ministry of Education, Xining 810000, China; zhangjintao138319@yeah.net (J.Z.); jinx13@lzu.edu.cn (X.J.); zhang1986lele@163.com (L.Z.); 20211028@qhnu.edu.cn (K.D.); jinyx13@lzu.edu.cn (Y.J.); yyongxiao@126.com (Y.Y.); 2 College of Geographical Sciences, Qinghai Normal University, Xining 810000, China; 3 Service and Support Center of Qilian Mountain National Park in Qinghai, Xining 810000, China; qhyuhy@163.com (H.Y.); 15796782519@163.com (X.W.)

**Keywords:** Qilian Mountains, marsh wetlands, microorganisms, soil, high-throughput sequencing

## Abstract

The microbial community is one of the key indicators for evaluating the health of alpine marsh wetlands, and understanding the composition and health of alpine wetland communities provides a scientific rationale for conservation and restoration efforts. Taking the alpine marsh wetlands in Qilian Mountain National Park, Qinghai Province, as the research object, 27 soil samples (0–30 cm depth) were collected in July 2024 from three types of wetlands: non-degraded (ND), low-level degraded (LD), and heavily degraded (HD). Using high-throughput sequencing, PICRUSt2 functional prediction, nonmetric multidimensional scaling (NMDS), and redundancy analysis (RDA), we analyzed the bacterial community structure and functional characteristics as well as the soil physicochemical properties across different degradation levels and soil depths. Pearson correlation analysis and RDA were used to identify key soil indicators influencing microbial community characteristics. The results showed that (1) compared to ND, the relative abundance of Acidobacteriota increased from 12.3% to 23.7%, and that of Pseudomonadota increased from 28.5% to 35.1% in HD wetlands. Meanwhile, the Shannon index rose from 5.31 in ND to 6.52 in HD, indicating significantly increased microbial community diversity and complexity with wetland degradation (*p* < 0.05). (2) Vertically, the six major primary metabolic functions gradually weakened with increasing soil depth in all three types of wetlands, the relative abundance of Proteobacteria decreased from 0 to 30 cm, and the α-diversity indices of soil bacteria also declined with depth. (3) Compared to ND, LD and HD showed significantly lower soil moisture content, organic matter, and total organic carbon (*p* < 0.05), while total potassium and pH increased significantly (*p* < 0.05). With increasing depth, total nitrogen significantly decreased across all degradation types (*p* < 0.05). Bacterial diversity, as measured by the Shannon and Simpson indices, showed a significant correlation with several soil properties (moisture, organic matter, total nitrogen, total potassium, cation exchange capacity, and total organic carbon; *p* < 0.05). Furthermore, pH emerged as a primary environmental driver shaping microbial community structure across different soil depths. These findings offer technical guidance and a theoretical framework for comprehending the degradation and restoration dynamics of alpine marsh wetland ecosystems in the Qilian Mountains.

## Simple Summary

This study investigates the changes in soil microbial communities across different degradation levels and soil layers in the alpine marsh wetlands of Qilian Mountain National Park, Qinghai. By systematically analyzing soil samples from non-degraded, low-level degraded, and heavily degraded wetlands, the study found that soil moisture and nutrient levels significantly declined with increasing degradation, while soil alkalinity increased. Meanwhile, the diversity and complexity of soil microbial communities showed an upward trend. The results highlight that changes in soil conditions deeply influence microbial community structure. This study sheds light on the ecological mechanisms driving wetland degradation. By doing so, it establishes a scientific basis for effective conservation and restoration strategies in alpine wetlands, ultimately fostering ecosystem stability and promoting sustainable development.

## 1. Introduction

Alpine marsh wetlands refer to a unique type of wetland ecosystem located in high-latitude or high-altitude regions [1], and are widely distributed across the Qinghai-Tibet Plateau, the Himalayas, and the Mongolian Plateau. These wetlands are characterized by typical cold-climate features, such as low annual average temperatures and limited precipitation [2]. However, due to the presence of permafrost layers that prevent water infiltration and the low evaporation rates, the surface remains perennially moist, forming marshy landscapes. Alpine marsh wetlands play an irreplaceable role in maintaining regional ecological balance. They not only effectively conserve water resources [3], purify water, and sustain the carbon cycle, but also provide vital habitats for many endemic and rare species of the plateau. Their unique and comprehensive ecological service functions are of great significance to the plateau ecosystem [4,5]. For instance, studies have shown that these wetlands contribute up to 30% of total water recharge in headwater basins and store significant amounts of soil organic carbon—estimated at over 40 kg/m^2^ in undisturbed sites [6]. This highlights their role not only in hydrological regulation but also in climate change mitigation. In recent years, however, factors such as global warming and increasing human disturbance have led to varying degrees of degradation in these wetlands. According to Zhang et al. (2024), alpine wetlands on the Qinghai-Tibet Plateau have decreased by over 10% in area over the past three decades due to permafrost degradation and warming-induced hydrological changes [3]. This widespread degradation poses a serious threat to ecological stability and water retention capacity in these regions. Manifestations include disrupted hydrological conditions, increased carbon emissions, and reduced ecosystem stability [7], highlighting the growing ecological vulnerability of alpine marsh wetlands. Therefore, assessing the health of degraded marsh wetlands is crucial for accurately identifying the extent of eco-logical function loss and degradation, and offers a scientific foundation for focused resto-ration efforts and effective management strategies.

Traditional assessments of marsh wetland health have primarily focused on core indicators such as soil, vegetation, and hydrology, exploring the ecological functions and degradation trends of wetlands from a macro perspective [8,9]. However, with advances in research methods, an increasing number of studies have begun to incorporate biological attributes at the micro level to better understand the internal processes and health status of marsh wetland ecosystems. For instance, Abulaizi et al. (2023) found that the bacterial richness index (Chao1) in non-degraded alpine wetlands was 18.7% lower than that in degraded sites, indicating that microbial parameters can sensitively reflect degradation stages [4]. Soil microorganisms are vital to wetland ecosystems [10,11], where they are crucial for organic matter decomposition [12] and the regulation of carbon and nitrogen cycling. Moreover, changes in dominant microbial taxa, diversity in-dices, and functional gene expression can provide rapid and sensitive indicators of wet-land health and functional stability.

Current research on marsh wetland soil microorganisms primarily focuses on analyzing spatial heterogeneity in the horizontal direction [13,14,15], with relatively few studies exploring vertical differentiation patterns of microbial communities within soil profiles. Situated at the convergence of temperate and frigid climate zones, Qilian Mountain National Park experiences a temperate arid climate in its southern region and a temperate semi-arid climate in its northern part [16,17]. In high-altitude regions—especially areas above 4000 m—cold climate characteristics become prominent due to low temperatures. This unique geographical position and vertical climatic gradient have fostered a diverse and complex ecosystem, with alpine marsh wetlands forming a particularly prominent high-altitude wetland cluster. Recognizing the crucial role of these Qilian Mountain wetlands in water conservation, supplying the Heihe and Shiyang Rivers, and acting as an ecological buffer between the Qinghai-Tibet Plateau and arid zones [18,19], this study investigates alpine marsh wetlands within the Qilian Mountain National Park that exhibit varying degrees of degradation. Focusing on the top 30 cm of soil, this research seeks to analyze how soil physicochemical properties and bacterial community structures vary both horizontally and vertically across different degradation levels and with soil depth. The ultimate goal is to establish a theoretical foundation and provide technical guidance for the effective evaluation and conservation of alpine marsh wetlands in the Qilian Mountains.

## 2. Materials and Methods

### 2.1. Overview of the Study Area

**Figure 1:**
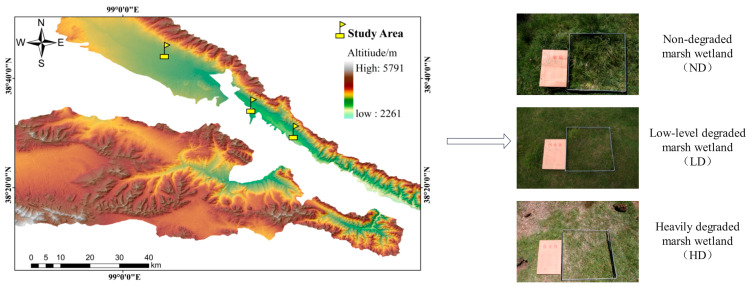
Overview map of the study area and alpine marsh wetlands with different degradation levels. The red Chinese characters on the folder (“档案袋”) in the image refer to a field sample documentation envelope, and the handwritten text indicates the sample ID used during fieldwork. These labels are for internal recording purposes only and do not affect the interpretation of the image.

The research was conducted within the Qilian Mountain National Park, situated in Qilian County, Haibei Tibetan Autonomous Prefecture, Qinghai Province (Figure 1). This region, with an average elevation of 3500–4200 m, has a plateau continental climate marked by substantial daily temperature fluctuations, intense solar radiation, cold, dry winters, and brief, cool summers [20]. With a long-term average temperature falling below −2.8 °C, the area also receives an annual average precipitation of 280.5 mm, with the majority falling as orographic rain between July and September. The dominant soil type is al-pine meadow soil. Major plant species in the study area include *Kobresia schoenoides*, *Blysmus sinocompressus*, *Kobresia humilis*, and *Polygonum viviparum*.

### 2.2. Plot Design and Sample Collection

A field survey was conducted in the study area in July 2024. The classification of wetland degradation levels was based on the Classification Standard for Degradation Levels of Alpine Marsh Wetlands in Qinghai Province (DB63/T 1794-2020), combined with on-site field survey data. Quantitative indicators included vegetation coverage, surface water coverage, and the number of rodent burrows. Specifically, ND (non-degraded) sites had vegetation coverage greater than 80%, surface water coverage exceeding 60%, and no more than one rodent burrow per 100 m^2^. LD (low-level degraded) sites had vegetation coverage of 50–80%, surface water coverage of 30–60%, and two to four rodent burrows per 100 m^2^. HD (high-level degraded) sites had vegetation coverage below 50%, surface water coverage less than 30%, and more than five rodent burrows per 100 m^2^. These thresholds were adapted based on regional experience and adjusted according to the actual conditions observed in the field [21]. Based on these criteria, the study plots were categorized into three degradation levels: non-degraded marsh wetland (ND), low-level degraded marsh wetland (LD), and heavily degraded marsh wetland (HD) (Table 1). For each plot, three replicate quadrats (100 cm × 100 cm) were randomly established [22], with a minimum spacing of 100 m between replicates. Following a five-point sampling pattern [23], from each soil profile, three soil cores were collected using a 100 cm^3^ auger and subsequently divided into three depth sections: 0–10 cm, 10–20 cm, and 20–30 cm. At each soil depth, three replicate subsamples were collected and evenly mixed to form a composite sample, which was then divided into three technical replicates for downstream analysis. Statistical analysis was based on the composite samples. The total soil samples were weighed and labeled. One portion was air-dried, cleaned of impurities (rocks, plant roots, dead leaves) [24], ground, and sieved for soil physicochemical property analysis, while the remaining portion was placed into sterile 20 mL EP tubes and kept in liquid nitrogen for subsequent analysis high-throughput microbial sequencing by Shanghai Meiji Biotechnology Co., Ltd. (Shanghai, China).

### 2.3. Soil Physicochemical Properties

Soil water content in each plot was measured using a portable time-domain reflectometry (TDR) soil moisture sensor. Soil pH was determined with a calibrated pH meter [25]. Total nitrogen (TN) was quantified following the Kjeldahl digestion protocol as specified in HJ 717-2014 [26]. Total phosphorus (TP) was assessed using the alkali fusion-molybdenum antimony colorimetric method (HJ 632-2011), while total potassium (TK) concentrations were measured by flame atomic absorption spectrophotometry (NY/T 87-1988) [27]. Soil organic matter (SOM) content was determined using the potassium dichromate–sulfuric acid oxidation method [28]. Cation exchange capacity (CEC) was analyzed via the cobalt hexamine trichloride cold-extraction method combined with spectrophotometry (HJ 889-2017). Total organic carbon (TOC) was evaluated using the combustion oxidation method with non-dispersive infrared detection (HJ 695-2014) [29].

### 2.4. Soil Microbial DNA Extraction and Gene Analysis

Isolation of soil microbial genomic DNA was done according to the protocol of the E.Z.N.A.^®^ Soil DNA Kit (Omega Bio-tek, Norcross, GA, United States). The extracted DNA was analyzed for concentration, purity (A260/A280) and integrity using the first-dimensional gel electrophoresis (1% with agarose and NanoDrop 2000 micro-spectrophotometer (Thermo Scientific, Waltham, MA, United States). Then, an An V3–V4 region of the bacterial 16S rRNA gene was specifically amplified by PCR with barcoded universal primers 338F and 806R, the buffer, dNTPs, the forward and reverse primers, DNA polymerase and the template DNA. Finally, a PCR carried out on The PCR products obtained was resolved on 2% agarose gel and the target bands were excised from the gel and purified with the help of PCR Clean-Up Kit (YuHua, Shanghai, China). The quantity of the purified DNA was measured precisely using Qubit 4.0 fluo-rometer (Thermo Scientific, Waltham, MA, United States). Library preparations were performed using NEXTFLEX^®^ Rapid DNA-Seq Kit (PerkinElmer, Waltham, MA, United States) and sequencing of samples was completed using paired-end sequencing (PE250) on an Illumina NextSeq 2000 platform.

### 2.5. Data Processing

Raw paired-end sequencing data were processed for quality control using fastp (v0.19.6). In this step, bases with Phred scores below 20 at the read ends were trimmed, and reads were truncated using a sliding window of 50 bp when the average quality in the window dropped below 20. Sequences shorter than 50 bp after trimming or containing ambiguous nucleotides (N) were excluded. High-quality reads were then merged with FLASH (v1.2.11), requiring a minimum overlap of 10 bp and allowing a mismatch rate of up to 20% within the overlap region. Merged reads were demultiplexed based on barcodes and primer sequences, with strict criteria—no mismatches allowed in barcodes and up to two in primers. To eliminate artifacts, chimeric sequences were detected and removed using UPARSE (v7.1), and OTUs were generated at 97% sequence identity.

Taxonomic annotation was performed using the Greengenes reference database, and the OTU abundance table was normalized by the number of reads per sample. For statistical evaluation of diversity and environmental factors among degradation levels, one-way ANOVA was conducted. Alpha diversity metrics, including ACE, Chao1, Shannon, and Simpson indices, were calculated. Relationships between alpha diversity and soil physicochemical parameters were assessed using Pearson correlation. Beta diversity, reflecting variations in microbial community structure, was visualized via non-metric multidimensional scaling (NMDS). Functional inference and metabolic pathway prediction were carried out with PICRUSt2. Redundancy analysis (RDA) was employed to explore how environmental variables influenced microbial assemblages across the 0–30 cm soil profile under different degradation intensities. Data visualization was conducted using R (v4.3.1) and Origin 2021, while Microsoft Excel assisted with data organization. Statistical significance was assessed using ANOVA in IBM SPSS Statistics 27 with Duncan’s post hoc test.

## 3. Results

### 3.1. Microbial Community Composition and α-Diversity Characteristics in the 0–30 cm Soil Layer of Marsh Wetlands

**Figure 2:**
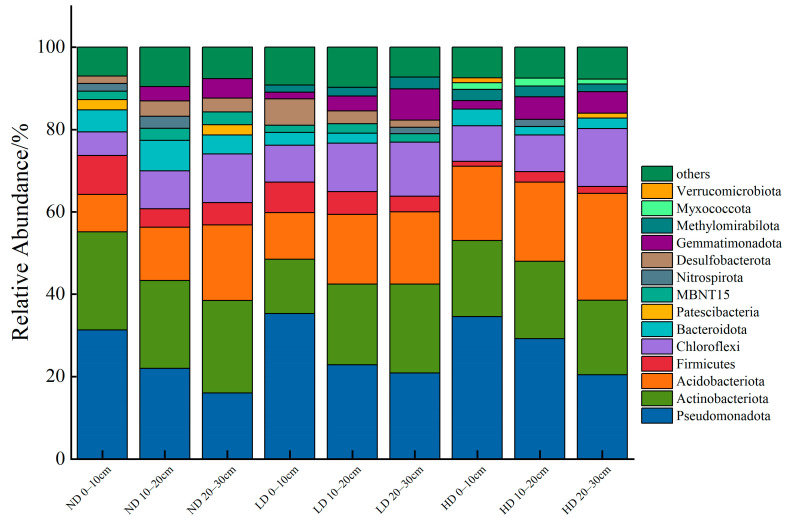
Composition of dominant soil bacterial communities in alpine swamp wetlands under varying degradation levels within the 0–30 cm soil layer.

High-throughput sequencing of the 16S rRNA gene from Bacteria, with 97% similarity, detected a diverse community belonging to 62 phyla, 188 classes, 467 orders, 742 families and 1360 genera. Pseudomonadota, Actinobacteriota, and Acidobacteriota were the most abundant, contributing to more than 55% in the total microbial community and peak at 71.12% in the highly degraded (HD) 0–10 cm soil layer (Figure 2). With increasing degradation, the relative abundance of Acidobacteriota significantly increased across the three types of marsh wetlands (*p* < 0.05). Compared to ND, the average relative abundance of Acidobacteriota in HD increased by 56.54%. Vertically, the relative abundance of Pseudomonadota gradually decreased within the 0–30 cm soil layers of all three wetland types. Compared to the surface soil (0–10 cm), the relative abundance of Pseudomonadota in the deep soil (20–30 cm) decreased by 48.85%, 41.00%, and 40.98% in ND, LD, and HD, respectively.

Soil microbial samples that fell under 0–30 cm affiliated to different types of degradation in alpine marsh wetlands had its diversity indices calculated as shown in the Table 2. Ace and Chao indices were applied to estimate the richness of the soil bacterial communities while the Shannon and Simpson index reflected on the diversity of the bacteria. As can be seen in Table 2, there was a decreasing trend of Ace and Chao indices of ND, LD, and HD sites with increased soil depth suggesting that total bacterial species richness declined on an overall basis deeper in the soil. In the same way, the Simpson index decreases as the soil layers become deeper. However, the overall diversity indices of ND, LD, and HD indicate that when comparing among the levels of degradation, the richness and diversity of the soil bacteria community also increase with the level of degradation in the soil of the 0–30 cm soil horizon.

### 3.2. Analysis of Microbial Community Beta Diversity in Marsh Wetlands

**Figure 3:**
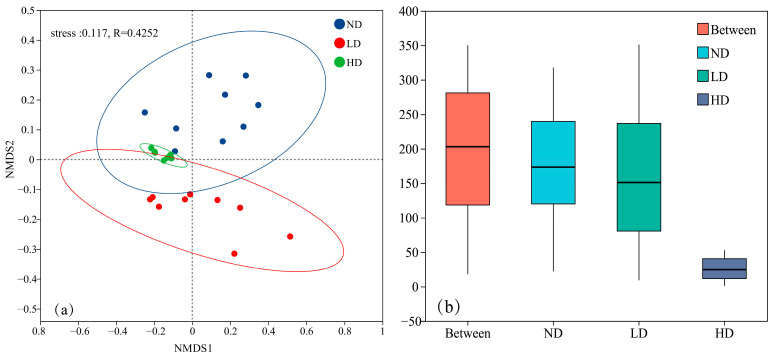
NMDS analysis and intergroup difference analysis of soil microbial communities in different types of degraded swamp wetlands; (**a**) NMDS plot based on Bray–Curtis dissimilarity at the OTU level. The x and y axes represent NMDS1 and NMDS2 dimensions, respectively. Stress = 0.117; (**b**) ANOSIM analysis.

Beta diversity of microbial communities in ND, LD and HD marsh wetlands was considered with the help of non-metric multidimensional scaling (NMDS) using the Bray–Curtis algorithm. The produced NMDS plot (Figure 3a) had a stress level of 0.117 (stress < 0.2) that implied that the obtained analysis had a reliable representation of the actual patterns. To confirm the differences presented in the soil microbial community composition between the three different types of wetland, ANOSIM (Analysis of Similarities) was also performed (Figure 3b). The *x*-axis represents the intergroup (Between) and intragroup samples, while the *y*-axis denotes the rank of distance. The median rank similarity between groups was higher than that within groups, indicating that intergroup differences were greater than intragroup differences (*p* < 0.001).

### 3.3. Functional Profiling of Soil Microbial Communities in Marsh Wetlands Across Degradation Gradients

Using the PICRUSt2 functional prediction tool, the potential functional roles of soil microbial communities within the 0–30 cm soil layer of alpine marsh wetlands exhibiting varying degradation levels were inferred by comparing microbial 16S rRNA gene sequences with the KEGG database. As shown in Table 3, six primary metabolic pathways were found in the soil bacteria of the three wetland types (ND, LD, and HD). Among them, metabolism was the dominant function, accounting for 77.10% to 78.73% of the total predicted functions, while organismal systems had the lowest proportion, ranging from 1.74% to 1.84%. As soil depth increased, the relative abundance levels of metabolism and genetic information processing functions showed an upward trend across the three degradation types, whereas the proportions of the other four functional categories gradually declined.

### 3.4. Impact Characteristics of Degraded Alpine Marsh Wetlands on Soil Physicochemical Properties

**Figure 4:**
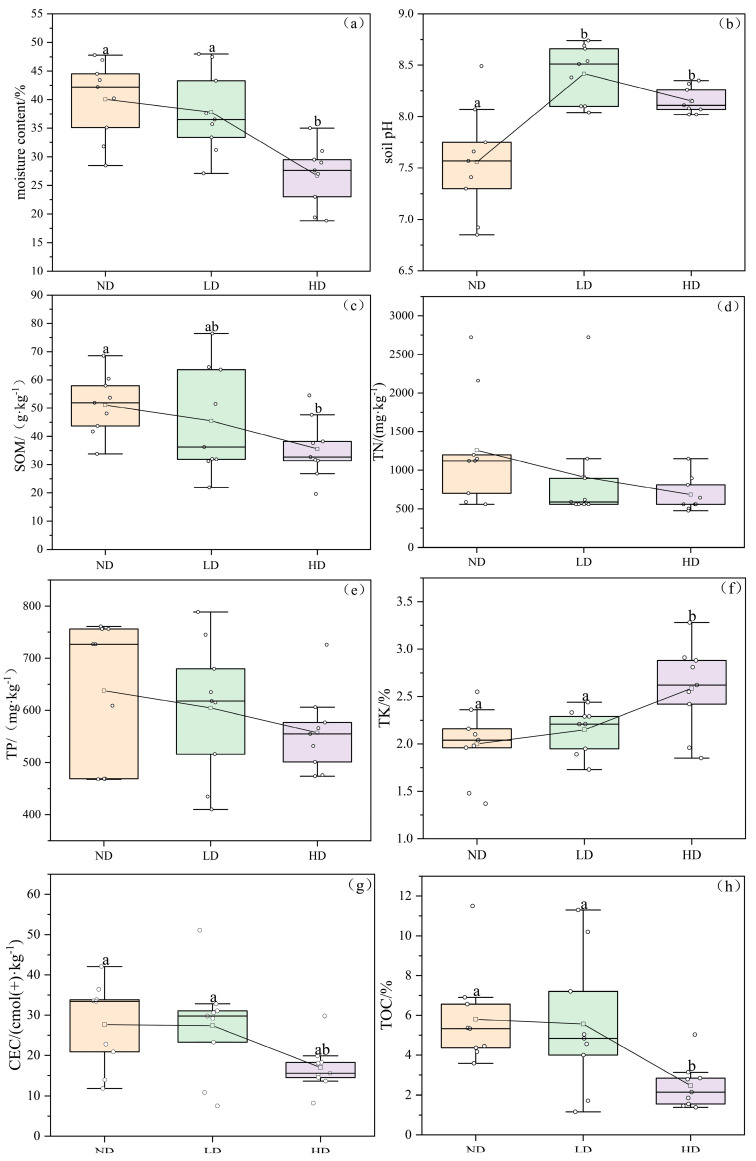
Differences in soil physicochemical properties under different degrees of degradation in alpine swamp wetlands. (**a**) Soil moisture content; (**b**) Soil pH; (**c**) Soil organic matter (SOM); (**d**) Total nitrogen (TN); (**e**) Total phosphorus (TP); (**f**) Total potassium (TK); (**g**) Cation exchange capacity (CEC); (**h**) Total organic carbon (TOC). Different lowercase letters indicate significant differences among alpine marsh wetlands with varying degradation levels (*p* < 0.05); same below.

The content from the soil moisture showed a decreasing trend with increasing degradation levels of marsh wetlands (Figure 4), with the most significant difference observed between ND and HD (*p* < 0.05). As degradation progressed, soil pH initially showed an initial increase followed by a decrease. This trend was observed along the degradation gradient of marsh wetlands, SOM, TN, TP, CEC, and TOC all exhibited declining trends. However, the variations in TN and TP among the three types of wetlands were not significant. In contrast, TK content was the lowest in non-degraded marsh wetlands and the highest in heavily degraded wetlands, showing an increase of 19.36%.

**Figure 5:**
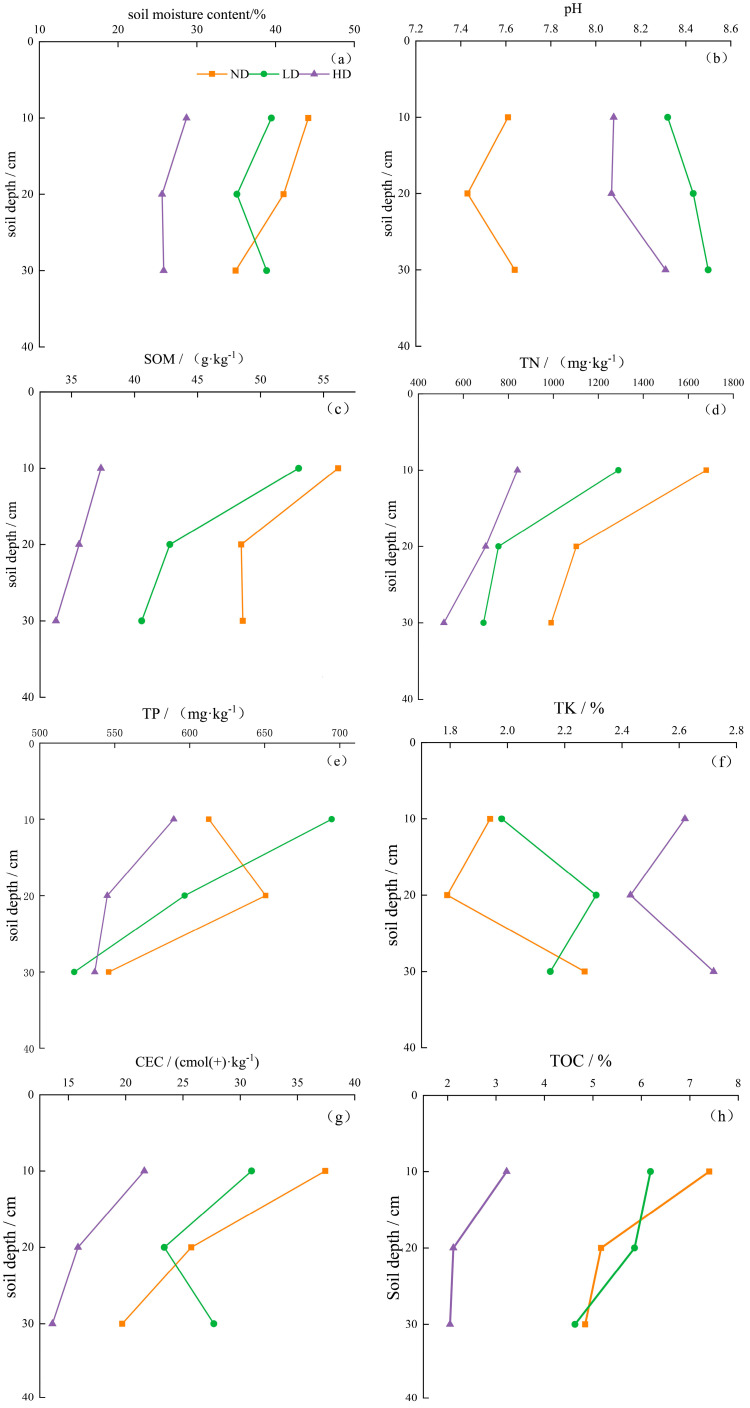
Variation in total organic carbon content with soil depth under different wetland degradation levels. (**a**) Soil moisture content; (**b**) Soil pH; (**c**) Soil organic matter (SOM); (**d**) Total nitrogen (TN); (**e**) Total phosphorus (TP); (**f**) Total potassium (TK); (**g**) Cation exchange capacity (CEC); (**h**) Total organic carbon (TOC).

Figure 5 illustrates that in heavily degraded wetlands, soil water content, SOM, TN, TP, CEC, and TOC all tended to decrease as soil depth increased. In non-degraded wet-lands, soil organic matter, TN, TP, CEC, and TOC generally showed a decreasing trend across the 0–30 cm soil profile. However, TP content slightly increased at the 10–20 cm layer, followed by a sharp decline at 20–30 cm, with a 16.08% decrease compared to the 10–20 cm layer. TN content exhibited a consistent decreasing trend across the 0–30 cm soil layers in all three degradation types, with the most significant decline observed in ND (*p* < 0.05).

## 4. Discussion

### 4.1. Variation in Soil Physicochemical Properties Across Degradation Levels and Soil Depths in Alpine Marsh Wetlands

As marsh wetland degradation intensifies, soil moisture content generally decreases. This trend may be because wetland degradation often involves a loss of vegetation cover, which reduces the soil’s water retention ability and enhances evaporation, ultimately leading to direct moisture loss through evaporation and diffusion [30]. Simultaneously, the exposure of soil surfaces can result in direct damage to soil structure due to increased solar radiation and wind force. This is mainly reflected in the decreased ratio of capillary to non-capillary porosity, which leads to enhanced water permeability and aeration, making it easier for water to be lost through surface runoff or deep infiltration [31]. Regardless of whether the marsh wetland is degraded or not, the soil pH remains alkaline. However, degraded wetlands exhibit higher pH values than non-degraded ones. This may be because the bedrock in the Qilian Mountain region is primarily composed of sedimentary rocks (such as limestone and dolomite), which release alkaline substances during weathering. These substances are then transported by surface runoff or groundwater into the wetlands, thereby increasing the soil pH. During the transition from non-degraded to degraded wetland, enhanced aeration accelerates the decomposition of organic acids in the soil, further raising the pH value [32].

Soil organic matter (SOM) is a key nutrient in wetland ecosystems, generally formed by the accumulation of plant and animal residues as well as microbial metabolites in the soil. It plays a crucial role in the ecosystem services and long-term stability of marsh wet-lands [6]. From the non-degraded (ND) to heavily degraded (HD) stages, SOM content shows a gradual decline. This is primarily due to the transition of wetlands from long-term anaerobic, waterlogged conditions to a more oxidized state, which accelerates the decomposition of organic matter by aerobic microorganisms [33]. Organic matter surfaces possess numerous negatively charged sites, such as carboxyl groups, which exhibit strong adsorption capacity for cations. Thus, the decrease in SOM content directly affects the variation in cation exchange capacity (CEC) in marsh wetland soils. These organic substances also include nitrogen-containing compounds, leading to a gradual reduction in total nitrogen (TN) content across ND, LD, and HD stages. Additionally, the decline in plant community abundance and weakening of biological nitrogen fixation capabilities further exacerbate the reduction in TN content. As the surface soil of marsh wetlands directly receives inputs of microbial residues and plant litter—making it the most active zone for nutrient transformation and accumulation—and given the water-saturated, oxy-gen-limited wetland environment, nutrient decomposition occurs more slowly, which favors nutrient retention. As a consequence, higher concentrations of SOM, TN, TP, and TK were primarily observed in the upper 0–10 cm of the 0–30 cm soil layer across all three wetland types. Moreover, the abundant microbial residues and plant litter in the surface soil release a greater amount of organic acids during decomposition, resulting in lower pH values in the 0–10 cm layer compared to the 20–30 cm layer.

In non-degraded marsh wetlands, iron (Fe) typically exists in its reduced form as Fe^2^⁺, which can combine with phosphate to form insoluble compounds such as vivianite (e.g., Fe_3_(PO_4_)_2_·8H_2_O) [34]. However, during the degradation of marsh wetlands, the originally anaerobic conditions gradually shift to oxidizing environments, leading to the oxidation of Fe^2^⁺ to Fe^3^⁺ and the formation of iron oxides (e.g., FeO(OH)) [35]. These oxides have smaller specific surface areas and altered surface charges, significantly reducing their ability to adsorb phosphate. Consequently, total phosphorus (TP) content in the soil de-creases with increasing wetland degradation. At the same time, such oxidative environments accelerate the mineralization of organic matter, resulting in the decomposition of humus and plant and animal residues into CO_2_, thereby reducing the total organic carbon (TOC) content in the soil. Unlike SOM, CEC, TOC, TN, and TP, the total potassium (TK) content increases with wetland degradation. This is primarily due to the enhanced weathering processes in degraded wetlands, which accelerate the release of potassium from potassium-bearing minerals [36]. Additionally, the reduction in vegetation communities in degraded wetlands slows the biological uptake of potassium, causing it to accumulate in the soil. This result corresponds with the findings reported by Zhang, M. et al. [10]. In summary, the varying pH conditions, soil moisture levels, and nutrient environments under different wetland degradation stages provide heterogeneous habitats for soil microbial communities.

### 4.2. Microbial Community Structure in Relation to Soil Factors and Their Interactive Dynamics

In the three degradation types of alpine swamp wetlands, the top three dominant bacterial phyla are Pseudomonadota, Actinobacteriota, and Acidobacteriota. As the soil depth increases from ND, LD, to HD, the TN content gradually decreases. Many groups within the Pseudomonadota phylum are nitrogen-demanding microorganisms [37]. The reduction in TN content limits the availability of absorbable nitrogen, which in turn inhibits the metabolism and energy synthesis of these nitrogen-demanding microbes. Consequently, the abundance of these microbes shows a strong positive correlation with the total nitrogen (TN) content in the soil layers. Furthermore, compared to the deeper soil layers (20–30 cm), the surface soils have higher oxygen levels and more easily degradable carbon sources such as sugars and amino acids [38], which provide favorable metabolic conditions for Pseudomonadota. In contrast, the deeper soil layers, with limited oxygen and higher amounts of recalcitrant organic matter, reduce the metabolic advantages of Pseudomonadota. Concurrently, the decline in soil organic matter (SOM) with increasing depth further inhibits the metabolic activity of Pseudomonadota, leading to a reduction in both their population size and activity. This leads to a decrease in nitrogen mineralization and conversion rates, thereby contributing to the reduction of TN content. Thus, nitrogen deficiency in the deep soils of swamp wetlands and the changes in microbial communities create a feedback loop, exacerbating the simultaneous decline of Pseudomonadota and TN content.

Acidobacteriota exhibited a higher relative abundance in more severely degraded swamp wetlands. Previous studies have indicated that Acidobacteriota thrive in slightly acidic to neutral soil environments [39]. Their relative abundance has been shown to be negatively correlated with soil pH in swamp wetlands. In the current research, as the wetland went through a process of degradation, the pH of the soil increased, but the relative abundance of Acidobacteriota increased with degradation. This result is inconsistent with previous findings and may be explained by two factors: (1) In degraded wetlands, the decomposition rate of SOM accelerates, leading to a reduction in easily decomposable carbon sources and an increase in recalcitrant carbon sources such as humus. As oligotrophic microorganisms [40], Acidobacteriota may have a competitive advantage in environments with complex carbon sources; and (2) although the overall soil pH tends to become more alkaline, the presence of microenvironments such as humus particles—particularly humic aggregates in degraded swamp wetland soils—can create localized acidic niches. These microscale environments not only buffer the pH fluctuations caused by alkaline conditions but also provide Acidobacteriota with electron donors and recalcitrant carbon sources [41], offering favorable refugia for their survival. This phenomenon reflects the complexity of microbial adaptation strategies in swamp wetland soils and highlights the unique environmental context shaped by degradation processes.

**Figure 6:**
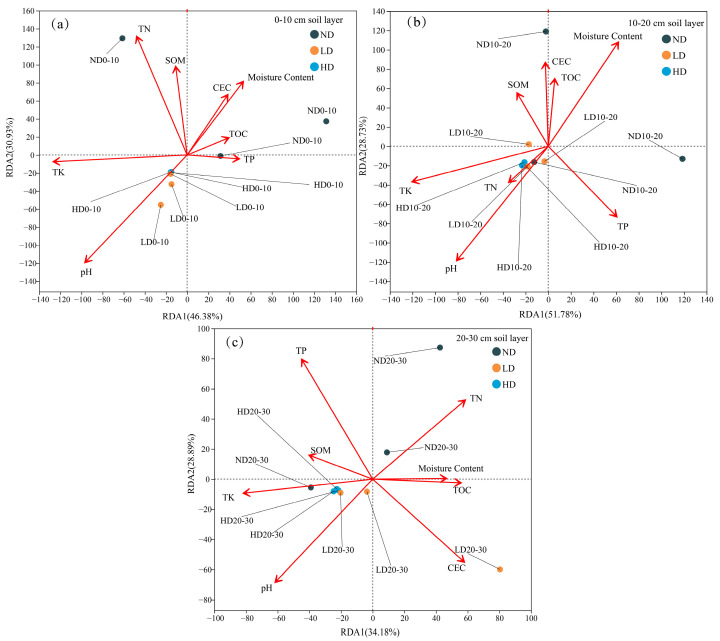
Redundancy analysis illustrating the relationships between soil properties and microbial community composition across three degradation levels of swamp wetlands. (**a**) 0–10 cm soil layer; (**b**) 10–20 cm soil layer; (**c**) 20–30 cm soil layer.

Soil physicochemical properties and their relationship to the structure of the microbial community in the 0–30 cm soil profile within three types of degraded alpine swamp wetlands were investigated using redundancy analysis (RDA). Soil physicochemical variables were found to explain an important amount of the variation in the microbial communities: 63.07% and 80.51% in the 0–10 cm layer and the 10–20 cm layer, and 77.31% in the 20–30 cm layer, respectively (Figure 6). These values represent the combined explanatory power of the first two RDA axes (RDA1 + RDA2) in each depth layer. Noteworthy, soil pH was a main feature in regard to the composition of microbial community in lightly and heavily degraded wetlands on the 0–10 cm (Figure 6a). For depths of 10–20 cm (Figure 6b), significant influence of the community structure of microbes was observed at different wetlands (ND, LD, and HD), by soil moisture content (*p* = 0.041) and pH (*p* = 0.021). On the other hand, not a single one of the environmental factors was making a significant contribution to the spatial pattern of microbial community structure through the 20–30 cm layer of the three wetland types (all *p* > 0.05) (Figure 6c). Overall, however, it was found that pH was the most potent environmental factor that regulated the structure in changes of microbial community in whole soil verticals with the most dominant trend in the 10–20 cm soil layer. As in the previous studies [42,43], pH can influence nutrient availability such as SOM and TOC, and thus the bacterial community structure. Moreover, CEC, TN, TP are influenced by SOM. Therefore, change in pH can control nutrient release and extraction of metal cations in swamp wetland soils manifesting overall through to the determining soil bacterial metabolic activity and ultimately distribution and structure. Moreover, beyond the control of soil pH by the microbial metabolic activities, these activities will directly influence the soil pH like in the decomposition of organic matter in these wetlands leading to increased acidity.

Table 2 presents the alpha diversity indices for soil microbial communities across different degradation levels of alpine swamp wetlands. The Ace and Chao indices indicate that the richness of bacterial communities is higher in degraded wetlands compared to undegraded ones, which aligns with observations reported by Zhang M. et al. in their research on Carex tussock marsh ecosystems [11]. Similarly, the Shannon and Simpson indices show greater bacterial community diversity in degraded wetlands. Two potential explanations for this observation are proposed, the first being the disturbance effect [44]. From LD to HD, the relative abundances of facultative bacteria and generalist bacteria, such as Pseudomonadota, Acidobacteriota, and Firmicutes, significantly increased. This suggests that although the original stable metabolic environment has been disrupted, degradation leads to an increase in the ecological niches and heterogeneity of the microbial environment in wetland soils [45], resulting in a temporary peak in diversity. However, as degradation continues, the richness and diversity of soil bacterial communities will gradually decrease, presenting a nonlinear response. Second, certain humus particles may retain more moisture, while degraded wetland areas are drier. These “microenvironments” provide survival opportunities for different types of soil bacteria, allowing bacteria adapted to different conditions to find their “niche”. Therefore, despite the overall environmental degradation, soil bacterial diversity may appear more diverse in the short term. However, this diversity reflects a signal that the swamp wetland ecosystem has lost its original balance and entered a state of turbulence.

**Figure 7:**
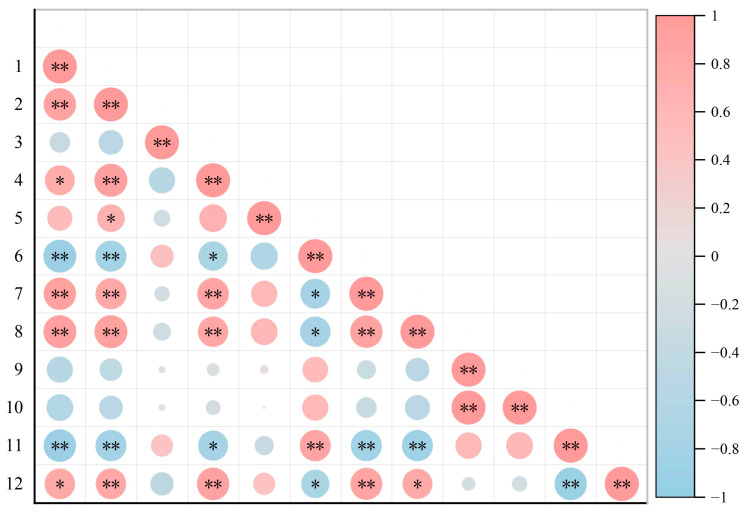
Associations between soil properties and microbial metrics across wetlands with varying degradation levels. The indicators include 1: soil moisture content, 2: SOM (Soil Organic Matter), 3: pH value, 4: TN (Total Nitrogen), 5: TP (Total Phosphorus), 6: TK (Total Potassium), 7: CEC (Cation Exchange Capacity), 8: TOC (Total Organic Carbon), 9: Ace index, 10: Chao index, 11: Shannon index, and 12: Simpson index. Asterisks indicate significant differences between the indicators of different degraded wetlands, with * representing *p* < 0.05 and ** representing *p* < 0.01. The color bar represents the scale of the correlation coefficient.

Pearson correlation analysis (Figure 7) was used to examine the relationships between soil microbial diversity and physicochemical properties. In particular, the soil bacteria Shannon index was found to be significantly negatively correlated with the soil water content, SOM, TN, CTC, and TOC (*p* < 0.05); however, it was quite positively correlated with TK (*p* < 0.01). Contrary to this, the Simpson index for soil bacteria presented strong correlations (*p* < 0.05) to positive with soil water content-SOM, TN, CEC, and TOC, whereas, with a negative correlation (*p* < 0.05) with TK. These findings represent different ways soil microbial community responds to different environmental factors. Widely used to characterize the diversity and stability of soil bacterial communities [46], the Shannon and Simpson indices showed very strong correlations with CEC and SOM content: terrible for Shannon (*p* < 0.01) and helpful for Simpson (*p* < 0.01). In undegraded swamp wetlands, the higher levels of organic matter and nutrients likely lead to a more balanced composition of soil bacterial populations, with a few dominant species adapted to resource-rich conditions. At this stage, the Shannon index is lower. However, in degraded swamp wetlands, the content of available nutrients decreases, nutrient competition intensifies, and environmental heterogeneity increases, which weakens the dominant position of previous dominant species. This promotes the coexistence of more generalist species. As a result, during this stage of degradation, the Shannon index significantly increases, reflecting an ecological succession response in which the soil microbial community structure shifts from “one dominant and many strong” to “multipolarization”.

Variations in soil microbial community structure, diversity index trends and physicochemical characteristics of soil with different levels of degradation and soil layers in high-altitude marsh wetlands were discussed in this research. On the microbiological level, it is intended to study the patterns and limitations of the degradation in the high-altitude marsh wetlands thus forming the directions for the degradation studying and restoration promoting of these wetlands.

## 5. Conclusions

(1) With the increasing degree of degradation, the soil moisture content, SOM, TN, TP, CEC, and TOC contents in the study area gradually decrease, while soil pH and TK content show an increasing trend. Moreover, with the increase in soil depth, SOM and TN contents show a gradual decrease in ND, LD, and HD, with the most significant decrease observed in ND. This indicates that the soil environment under different degradation levels and soil depths exhibits heterogeneous characteristics.

(2) As the degree of degradation deepens, differences in the composition of the soil bacterial community structure are observed. The relative abundance of Acidobacteriota significantly increases across the three types of degraded wetlands. The soil bacterial community richness and diversity show an overall upward trend in ND, LD, and HD. With increasing soil depth, the proportion of metabolism and genetic information processing functions shows an increasing trend in all three types of degraded wetlands.

(3) Concerning the types of wetlands, soil moisture content, SOM, TN, TK, CEC, and TOC are major environmental factors that influence diversity of soil bacterial community structure. In particular, pH was one of the most important environmental variables that influenced changes in structure of microbial communities across varying depths of soil.

These findings not only provide theoretical support for understanding alpine wetland degradation processes, but also offer a potential foundation for developing microbial indicators to guide ecological restoration and further explore the mechanisms of soil microbial community dynamics under environmental stress.

## Figures and Tables

**Table 1:** Basic information of the sample plot.

Degradation	Dominant Vegetation	Plot Overview
Non-degraded (ND)	*Kobresia schoenoides,* *Blysmus sinocompressus*	The area has abundant surface water, almost no dry patches, and a rich diversity of plant species
Low-level degraded (LD)	The abundance of wetland plants such as *Kobresia schoenoides* has decreased, while species like *Kobresia humilis* and *Kobresia capillifolia* have become more prevalent	Surface water is limited, with a few dry patches present, and the plant species are relatively diverse
Heavily degraded (HD)	Toxic species, including *Polygonum viviparum* and *Stellera chamaejasme* L., have become the dominant components of the plant community	The area lacks surface water, supports low plant diversity, exhibits a high density of rodent burrows, and shows evident signs of desertification

**Table 2:** Soil microbial diversity index of 0–30 cm soil layer in different degraded swamp wetlands.

Sample	Ace	Chao	Shannon	Simpson
ND0–10 cm	3449.04 ± 121.81 bc	3318.57 ± 64.75 bc	5.31 ± 0.17 d	0.0515 ± 0.0192 a
ND10–20 cm	3255.61 ± 277.06 c	3167.34 ± 272.91 bc	5.78 ± 0.29 c	0.0204 ± 0.0154 b
ND20–30 cm	2723.88 ± 94.68 d	2691.66 ± 77.7 d	5.90 ± 0.12 bc	0.0108 ± 0.0022 b
LD0–10 cm	3237.74 ± 353.28 c	3098.48 ± 296.6 cd	5.90 ± 0.36 bc	0.0209 ± 0.0247 b
LD10–20 cm	3212.51 ± 396.25 c	3112.44 ± 408.04 cd	6.00 ± 0.13 bc	0.0093 ± 0.0004 b
LD20–30 cm	2736.43 ± 359.29 d	2690.46 ± 338.46 d	5.79 ± 0.14 c	0.0146 ± 0.0107 b
HD 0–10 cm	4092.91 ± 120.22 a	3925.01 ± 73.91 a	6.52 ± 0.01 a	0.0044 ± 0.0002 b
HD10–20 cm	3629.70 ± 91.48 bc	3555.86 ± 57.30 ab	6.23 ± 0.07 ab	0.0059 ± 0.0007 b
HD20–30 cm	3833.48 ± 182.35 ab	3746.66 ± 132.50 a	6.36 ± 0.07 a	0.0053 ± 0.0005 b
ND	3142.84 ± 375.50 a	3059.19 ± 327.15 a	5.66 ± 0.31 a	0.0276 ± 0.0213 a
LD	3062.23 ± 282.43 a	2967.13 ± 239.70 a	5.90 ± 0.11 a	0.0149 ± 0.0058 a
HD	3852.03 ± 232.16 b	3742.51 ± 184.61 b	6.37 ± 0.15 b	0.0052 ± 0.0008 a

Note: Different lowercase letters (a–d) within each column indicate significant differences at *p* < 0.05.

**Table 3:** Main primary metabolic functions of microorganisms in the 0–30 cm soil layer of different degraded marsh wetlands.

Degradation Level	Primary Metabolic Functions					
	Metabolism	Genetic Information Processing	Environmental Information Processing	Cellular Processes	Human Diseases	Organismal Systems
ND0–10 cm	77.87%	6.74%	5.49%	4.58%	3.55%	1.77%
ND10–20 cm	78.26%	7.08%	5.12%	4.38%	3.38%	1.77%
ND20–30 cm	78.63%	7.34%	4.87%	4.28%	3.14%	1.74%
LD0–10 cm	77.10%	6.91%	5.65%	4.93%	3.57%	1.84%
LD10–20 cm	78.26%	7.21%	5.11%	4.45%	3.21%	1.76%
LD20–30 cm	78.30%	7.39%	4.96%	4.45%	3.16%	1.74%
HD0–10 cm	77.85%	6.90%	5.14%	4.51%	3.77%	1.84%
HD10–20 cm	78.03%	7.23%	4.98%	4.46%	3.51%	1.80%
HD20–30 cm	78.73%	7.34%	4.69%	4.20%	3.29%	1.75%

## Data Availability

The data presented in this study are available in the article and the original sequence data is stored in the NCBI database (PRJNA1265454).
